# Identification and Comparative Analysis of the Protocadherin Cluster in a Reptile, the Green Anole Lizard

**DOI:** 10.1371/journal.pone.0007614

**Published:** 2009-10-29

**Authors:** Xiao-Juan Jiang, Shaobing Li, Vydianathan Ravi, Byrappa Venkatesh, Wei-Ping Yu

**Affiliations:** 1 Gene Regulation Laboratory, National Neuroscience Institute, Singapore, Singapore; 2 School of Life Sciences, Shandong University, Jinan, China; 3 Institute of Molecular and Cell Biology, Agency for Science, Technology and Research (A*STAR), Singapore, Singapore; Michigan State University, United States of America

## Abstract

**Background:**

The vertebrate protocadherins are a subfamily of cell adhesion molecules that are predominantly expressed in the nervous system and are believed to play an important role in establishing the complex neural network during animal development. Genes encoding these molecules are organized into a cluster in the genome. Comparative analysis of the protocadherin subcluster organization and gene arrangements in different vertebrates has provided interesting insights into the history of vertebrate genome evolution. Among tetrapods, protocadherin clusters have been fully characterized only in mammals. In this study, we report the identification and comparative analysis of the protocadherin cluster in a reptile, the green anole lizard (*Anolis carolinensis*).

**Methodology/Principal Findings:**

We show that the anole protocadherin cluster spans over a megabase and encodes a total of 71 genes. The number of genes in the anole protocadherin cluster is significantly higher than that in the coelacanth (49 genes) and mammalian (54–59 genes) clusters. The anole protocadherin genes are organized into four subclusters: the δ, α, β and γ. This subcluster organization is identical to that of the coelacanth protocadherin cluster, but differs from the mammalian clusters which lack the δ subcluster. The gene number expansion in the anole protocadherin cluster is largely due to the extensive gene duplication in the γb subgroup. Similar to coelacanth and elephant shark protocadherin genes, the anole protocadherin genes have experienced a low frequency of gene conversion.

**Conclusions/Significance:**

Our results suggest that similar to the protocadherin clusters in other vertebrates, the evolution of anole protocadherin cluster is driven mainly by lineage-specific gene duplications and degeneration. Our analysis also shows that loss of the protocadherin δ subcluster in the mammalian lineage occurred after the divergence of mammals and reptiles. We present a model for the evolutionary history of the protocadherin cluster in tetrapods.

## Introduction

Since their discovery about a decade ago [1], [2], the vertebrate protocadherin cluster genes have received considerable attention due to their unusual genomic organization and potential role in specifying the remarkable diversity of the neural network. The clustered protocadherin genes are predominantly expressed in neurons and their protein products are highly enriched in synaptic junctions and axons [1], [3]–[5]. Single neuron RT-PCR experiments have demonstrated that individual neurons, even of the same kind, express an overlapping but distinct subset of protocadherin cluster genes [6]–[8]. Thus the combinatorial expression of protocadherins in individual neurons might provide a profound molecular code for specifying neuron-neuron connections in the developing nervous system [9]–[11]. Indeed, ablation of protocadherin α and γ subclusters in mice causes defects in axonal projection of olfactory sensory neurons to the olfactory bulb [12] or drastic impairment in synaptic formation and extensive loss of interneurons in the spinal cord [5], [13]. In mammals, the protocadherin cluster genes are organized into three closely-related subclusters, namely the α, β and γ subclusters, each of which contains 15 to 22 homologous “variable” exons that are arranged in tandem [2]. Each variable exon measuring about 2.4 kb is transcribed from an independent promoter and encodes an extracellular domain (comprising six calcium-binding ectodomain repeats), a transmembrane domain and a short segment of the intracellular domain. In addition to the variable exons, the 3′ end of the α and γ (but not β) subclusters contains three “constant” exons each, which are spliced to individual variable exons in their respective subclusters. These constant exons encode the major part of the intracellular domain. Thus, the protocadherin proteins produced by each of the α and γ subclusters comprise a homologous but distinct extracellular domain, and an identical cytoplasmic domain. The extracellular domain is presumably responsible for providing diverse signals for specifying cell-cell interaction through homophilic or heterophilic interaction [14], [15] or by interaction with other cell surface molecules [16], [17], whereas the cytoplasmic domain is likely to mediate a common intracellular process for implementing the cell interaction signal [18], [19]. The protein products encoded by the β subcluster genes, which lack the constant exons, contain only the diverse extracellular domain, and lack the common cytoplasmic domain [2].

The protocadherin cluster represents one of the most evolutionarily dynamic gene loci in vertebrate genomes. Comparative analysis of its subcluster organization and paralog arrangements has provided useful information regarding the dynamic nature of vertebrate genomes [20], [21]. To date, the genomic organization of protocadherin cluster has been characterized in several vertebrate lineages, including mammals [2], [22]–[25], chicken (the α subcluster only) [26], coelacanth [20], teleost fishes [27]–[29] and a cartilaginous fish, the elephant shark [21]. While the protocadherin cluster in mammals is organized into the α, β, and γ subclusters with 54 to 59 genes, the coelacanth cluster possesses an additional single-gene subcluster, the δ subcluster, at the 5′ end and consists of a total of 49 genes [20]. Teleost fishes such as fugu and zebrafish contain two unlinked protocadherin clusters, Pcdh1 and Pcdh2, due to a fish-specific genome duplication event. Both clusters lack the β subcluster. In addition, the fugu Pcdh1 cluster has lost the γ subcluster, thus containing only the δ and α subclusters. In contrast, the zebrafish Pcdh1 cluster has retained the δ, α and γ subclusters whereas the Pcdh2 cluster has lost the δ subcluster and retained only the α and γ subclusters [27]–[29]. The duplicate protocadherin clusters in fugu and zebrafish contain at least 77 and 107 genes, respectively. The elephant shark possesses three unique protocadherin subclusters in addition to the δ subcluster. These subclusters are designated as the ε, μ and ν subclusters. They have no orthologs in bony vertebrates [21]. The different subcluster complement in bony vertebrates and cartilaginous fishes suggests that the common ancestor of jawed vertebrates contained at least seven protocadherin subclusters (α, β, γ, δ, ε, μ and ν), of which the α, β and γ subclusters have been lost in the cartilaginous lineage, whereas the ε, μ and ν subclusters have been lost in bony vertebrates. The δ subcluster has been retained in elephant shark, teleost fishes, coelacanth, amphibians and birds [21], but lost in mammals. In addition to the loss of complete subclusters, the variable exons in each protocadherin subclusters (except the δ) has experienced repeated lineage-specific gene duplication and degeneration. For instance, while most human protocadherin cluster genes have a clearly-defined one-to-one ortholog in other mammals, only a few genes in the human and coelacanth clusters exhibit individual orthologous relationship, suggesting that the variable exons in each of the human and coelacanth clusters have experienced repeated lineage-specific gene duplication and degeneration [20], [22], [24]. Given the potential role of protocadherins in specifying the neural network, it is plausible that the high frequency of gene turnover in the protocadherin cluster might have played a key role in the adaptive evolution of the central nervous system in vertebrates. Among tetrapods, only mammalian protocadherin clusters have been fully characterized to date. Here, we report the identification and analysis of the protocadherin cluster in a reptile, the green anole lizard (*Anolis carolinensis*). The protocadherin cluster genes in anole, which represents an intermediate taxon between the coelacanth and mammals, fills a critical gap in the evolutionary history of the protocadherin cluster in tetrapods.

## Results and Discussion

### Anole protocadherin cluster consists of 71 genes, organized into δ, α, β and γ subclusters

**Figure 1:**
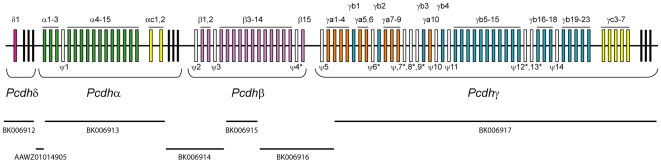
Genomic organization of the anole protocadherin cluster. Constant exons of the δ, α and γ subclusters are shown as black vertical bars at the end of each subcluster. Variable exons in the same paralog subgroup are indicated by the same color. Pseudogenes (ψ) are shown as open boxes. Sequence contigs corresponding to the anole protocadherin region are shown below the gene cluster.

To identify the protocadherin cluster sequence in the anole genome, we first performed a TBLASTN search against the draft anole genome (Broad Institute AnoCar 1.0) using amino acid sequences of mammalian protocadherin constant exons as queries. This led to the identification of a single scaffold (Scaffold_147, 2,899,420 bp) containing the entire protocadherin cluster. Inspection of this scaffold showed that the sequence corresponding to the anole protocadherin cluster represents a high-quality assembly region interrupted by 24 gaps. We subsequently filled 18 of these gaps by PCR amplification from genomic DNA resulting in seven contigs spanning ∼1 Mb. Annotation of this gene cluster by GENSCAN and homology comparisons identified 71 protocadherin variable exons and three subsets of constant exons (Fig. 1). We confirmed the splicing sites of the variable and constant exons by RT-PCR using cDNA from anole brain. In addition to the 71 intact variable exons, we were also able to identify 14 pseudogenes. Interestingly, half of these pseudogenes contain single-nucleotide insertion or deletion (Fig. 1). The presence of protocadherin pseudogenes at various stages of degeneration indicates that the protocadherin cluster has continued to experience gene losses in the anole lineage (see below). In addition to the protocadherin genes, we identified 19 non-protocadherin genes upstream and five non-protocadherin genes downstream of the protocadherin cluster. The synteny of these genes flanking the protocadherin cluster is almost totally conserved in the human protocadherin cluster locus (Table 1). This indicates that, in contrast to the protocadherin cluster, its flanking regions are highly stable in reptiles and mammals. The protocadherin clusters in human and mouse contain two non-protocadherin genes (*Slc25a2* and *Taf7*) located between the β and γ subclusters [2], [22]. However, these genes are not present either in the anole protocadherin cluster or in the protocadherin clusters of non-tetrapod vertebrates. Thus we conclude that these genes were inserted into the protocadherin cluster in the mammalian lineage after it diverged from reptiles.

**Table 1:** Conserved synteny in the anole and human protocadherin gene loci.

**5′ flanking genes**					
Gene description		Anole (*Anolis carolinensis*)		Human (*Homo sapiens*)	
		Ori	Size (kb)	Ori	Size (kb)
Nrg2	neuregulin 2 isoform3	−	>20.0	−	195.6
Pura	purine-rich element binding protein A	+	0.6	+	2.6
C5orf32	putative nuclear protein ORF1-FL49	+	14.7	+	68.7
Pfdn1	prefoldin subunit 1	−	36.4	−	58.1
Hbegf	heparin-binding EGF-like growth factor	−	5.0	−	13.7
Slc4a9	solute carrier family 4, sodium bicarbonate	+	52.1	+	14.8
Ankhd1	ankyrin repeat and KH domain containing 1	+	102.1	+	138.0
Eif4ebp3	eukaryotic translation initiation factor 4E	no homolog		+	1.9
Sra1	steroid receptor RNA activator 1	−	3.3	−	8.0
Apbb3	amyloid beta precursor protein-binding, family	−	13.1	−	6.3
Slc35a4	solute carrier family 34, member A4	+	1.8	+	4.3
Cd14	CD14 antigen precursor	−	0.9	−	1.7
Tmco6	transmembrane and coiled-coil domain 6	+	8.5	+	6.0
Ndufa2	NADH dehydrogenase 1 alpha	−	3.3	−	2.3
Ik	RED protein	+	11.3	+	14.7
Wdr55	WD repeat domain 55	+	7.0	+	5.9
Dnd1	dead end homolog 1	−	4.7	−	2.8
Hars	histidyl-tRNA synthetase	−	22.6	−	17.5
Hars2	histidyl-tRNA synthetase 2	−	23.0	+	7.9
Zmat2	zinc finger, matrin type 2	+	12.1	+	6.2
**3′ flanking genes**					
Gene description		Anole (*Anolis carolinensis*)		Human (*Homo sapiens*)	
		Ori	Size (kb)	Ori	Size (kb)
Diaph1	diaphanous 1 isoform 1	−	70.6	−	104.0
Hdac3	histone deacetylase 3	−	25.5	−	16.0
C5orf16	chromosome 5 open reading frame 16	+	10.5	+	4.0
Fchsd1	FCH and double SH3 domains 1	−	25.6	−	12.1
Centd3	centaurin delta 3	−	55.2	−	28.8

**Figure 2:**
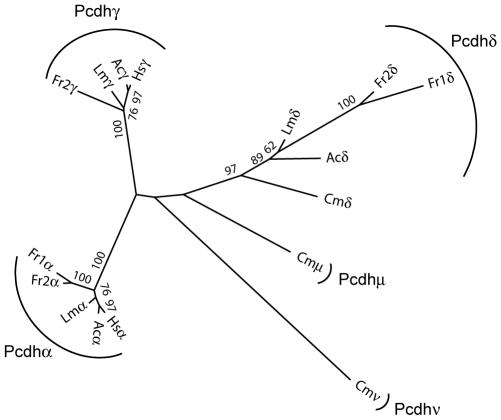
Phylogenetic analysis of protocadherin constant exon sequences. The phylogenetic tree was generated from alignments of protein sequences of the protocadherin constant regions by Maximum likelihood method using PhyML. Bootstrap values from 100 replicates are shown beside their respective branches. The tree is unrooted. Ac, *Anolis carolinensis*; Cm, *Callorhinchus milii*; Hs, *Homo sapiens*; Lm, *Latimeria menadoensis*; Fr, *Fugu rubripes*.

To determine the subcluster organization of anole protocadherin genes, we first performed phylogenetic analysis of the three subsets of constant exons from the anole protocadherin cluster together with constant exon sequences of protocadherin α, γ, δ, μ and ν subclusters from other representative vertebrates. The phylogenetic analysis shows that the three subsets of constant exons in the anole protocadherin cluster represent the δ, α and γ subclusters (Fig. 2). Since the protocadherin β subcluster lacks constant exons, the identity of this subcluster can only be inferred by the phylogenetic analysis of its variable exons. We therefore performed phylogenetic analysis of the variable exon sequences. This analysis showed that the 15 genes immediately downstream of the anole protocadherin α subcluster belong to the β subcluster (see below). Taken together, our results indicate that the anole protocadherin cluster consists of 71 protocadherin genes, which are organized into four subclusters: the δ (one gene), α (17 genes), β (15 genes) and γ (38 genes) (Fig. 1). The subcluster organization of the anole protocadherin cluster is therefore identical to that of the coelacanth cluster, but differs from the mammalian protocadherin cluster which lacks the δ subcluster at the 5′ end. Notably, the total number of genes in the anole protocadherin cluster (71 genes) is significantly higher than that in the coelacanth (49 genes) and mammalian (54–59 genes) clusters.

### Anole protocadherin genes have experienced a low frequency of gene conversion

It has been documented that protocadherin genes in teleost fishes and mammals have experienced repeated gene conversion events during evolution [27], [29]. In contrast, protocadherin genes in coelacanth and elephant shark have experienced only limited gene conversion events [21], [27]. To investigate whether the anole protocadherin genes have undergone gene conversion, we estimated the total number of synonymous substitutions per codon (dS) of the anole protocadherin genes in the four major paralog subgroups: *Acα1-15*, *Acβ1-15*, *Acγa1-10* and *Acγb4-23*. We used the synonymous substitution rate as a measure of the frequency of gene conversion because purifying selection for protein function does not act on synonymous sites. In case ECD5 and ECD6 domains of anole have experienced gene conversion, the synonymous substitution rate for these domains should be considerably lower than that for ECD1 to ECD4 domains. However, as shown in Table 2, the synonymous substitution rates in *Acα1-15*, *Acβ1-15* and *Acγb4-23* subgroups are highly similar among the six ectodomains. The ratios between the most and the least divergent ectodomains in these paralog subgroups range from 2.25 to 3.40, which are comparable to that of the coelacanth (1.59–1.75) [20] and elephant shark (1.8–2.3) [21] protocadherin paralog subgroups, but are significantly lower than that of zebrafish (79.5–1280) [27] and fugu (38.4 to >94.6) [29] paralog subgroups, suggesting that these anole protocadherin paralog subgroups have experienced little gene conversion. On the other hand, anole protocadherin subcluster *Acγa1-10* subgroup has a relatively higher ratio of 7.89 mainly because of the lower synonymous substitution rates in the ECD5 and ECD6 ectodomains. This suggests that only anole subgroup *Acγa1-10* has experienced a limited number of gene conversion events.

**Table 2:** Synonymous substitution rates^a^ of individual ectodomains of anole protocadherin subcluster genes.

Subgroups	dS_ECD1_	dS_ECD2_	dS_ECD3_	dS_ECD4_	dS_ECD5_	dS_ECD6_	dS_ECDhigh_/dS_ECDlow_ b
Acα1-15	0.151	0.370	0.513	0.259	0.162	0.232	3.40
Acβ1-15	0.139	0.179	0.126	0.160	0.144	0.071	2.52
Acγa1-10	0.294	0.374	0.513	0.433	0.168	0.065	7.89
Acγb4-23	0.227	0.223	0.256	0.222	0.145	0.114	2.25

a Average synonymous substitution per codon (dS) for each branch in the gene tree of individual subgroups was calculated based on the alignment of paralogs in the subgroup.

b The ratio of the average dS per branch calculated based on alignment of the most divergent (dS_ECDhigh_) and the least divergent (dS_ECDlow_) ectodomains in each protocadherin subgroup.

### Phylogenetic relationships of anole and other vertebrate protocadherin cluster genes

**Figure 3:**
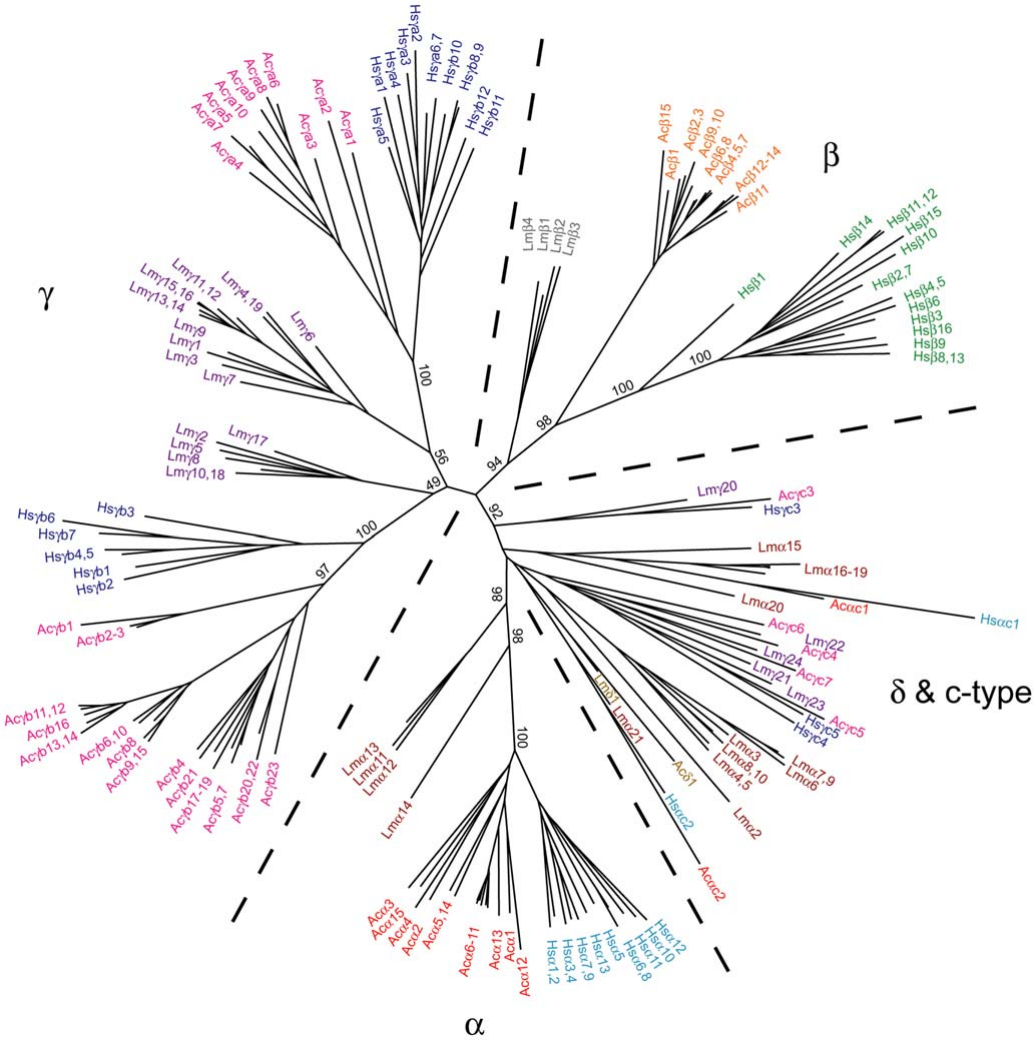
Phylogenetic analysis of protocadherin variable exon sequences. Protein sequences of the EC1-EC3 ectodomain region of anole, human and coelacanth protocadherin variable exons were aligned using ClustalW. The phylogenetic tree was generated by the Maximum likelihood method using PhyML. Protocadherin genes in the same paralog subgroups in different species are indicated by the same color. The robustness of the tree was determined using 100 bootstrap replicates. Bootstrap values for only the major branches are shown. The tree is unrooted.

Previous studies have shown that most mammalian protocadherin genes (*e.g.*, >72% in human and >67% in mouse) have clearly-defined one-to-one interspecies orthologous relationships [22], [24], [30]. However, few such orthologous relationships can be found between individual mammalian, coelacanth or teleost protocadherin genes. Instead, some of the mammalian protocadherin genes are orthologous to coelacanth and teleost protocadherin genes only as paralog subgroups [20], [27]–[29]. This type of phylogenetic relationships implies that subsequent to the divergence of vertebrate lineages, the variable exons of protocadherin clusters have undergone extensive gene turnover and the paralog complement in each of the current vertebrate protocadherin clusters is a result of multiple repeated lineage-specific gene duplication/degeneration events. To trace the evolutionary history of protocadherin genes in tetrapods, we performed phylogenetic analysis using individual variable exon sequences of anole, coelacanth and human protocadherin clusters. Coelacanth, which is the closest living relative of tetrapods whose protocadherin cluster has been characterized, was chosen as the outgroup. Our results show that the anole α subcluster consists of two divergent subgroups of protocadherin genes, the *Acα1-15* and the *Acαc1-2*. While *Acαc1* and *Acαc2* are clearly the anole orthologs of human *Hsαc1* and *Hsαc2*, respectively, the anole *Acα1-15* form a paralog subgroup on its own and is orthologous to the human paralog subgroup comprising *Hsα1-13* genes (Fig. 3). This phylogeny suggests that individual variable exons in each of the *Acα1-15* and *Hsα1-13* paralog subgroups are derived from a single ancestral protocadherin paralog in each of the anole and human α subclusters through multiple rounds of lineage-specific gene duplications, and the anole and human ancestral paralogs evolved from a single gene that existed in the common ancestor of reptiles and mammals. The relationships of the anole protocadherin genes to the coelacanth α subcluster however appear to be more complex. While it is clear that the last gene at the 3′ end of the coelacanth subcluster (*Lmα21*) is an ortholog of anole *Acαc2* and human *Hsαc2* (also located at the 3′ end of their respective subclusters), the coelacanth counterparts of anole *Acαc1* and human *Hsαc1* seem to have expanded into a paralog subgroup that contains six genes (*Lmα16-19*) (Fig. 3; also see Fig. S1 for a higher resolution phylogenetic tree for this class of protocadherin genes). It appears that the coelacanth protocadherin genes closest to the anole *Acα1-15* and human *Hsα1-13* paralog subgroups are the *Lmα14* and its closely related paralog subgroup *Lmα11-13*. Apparently, there is no equivalent to coelacanth *Lmα2-10* in anole and human α subclusters, suggesting that orthologs for these coelacanth genes have been lost in reptiles and mammals (Fig. 3). These results suggest that the paralog subgroup complement of the anole protocadherin α subcluster is highly similar to the human α subcluster, but considerably divergent from that of coelacanth protocadherin α subcluster.

The genomic organization of protocadherin β subcluster is relatively simple, containing only a single paralog subgroup and lacking the constant region [2], [30]. The protocadherin β subcluster has been identified only in mammalian and coelacanth protocadherin clusters, but not in fugu, zebrafish and elephant shark clusters, suggesting that it is specific to lobe-finned fishes and tetrapods. Our phylogenetic analysis shows that the first 15 protocadherin genes immediately downstream of the anole α subcluster, as a paralog subgroup, are orthologous to the human and coelacanth protocadherin β subcluster genes, indicating that this subset of anole protocadherin genes belong to the β subcluster (Fig. 3). The absence of one-to-one orthologous relationships between individual anole, human and coelacanth protocadherin β genes suggests that these genes were derived from multiple, independent lineage-specific gene duplication events in their respective subclusters. Thus, the evolution of protocadherin β subclusters is driven exclusively by lineage-specific variable exon duplication and degeneration. Notably, the gene number of the anole β subcluster (15 genes) is comparable to that of the human β subcluster (16 genes), but is significantly higher than that of the coelacanth β subcluster (4 genes). The expansion of β subcluster genes in reptiles and mammals might have given rise to a higher molecular repertoire to mediate a more diverse and/or complex cell-cell interaction network. However, as protocadherin molecules are highly homologous, and apparently redundant [31], whether the differential gene numbers of the β subcluster could indeed affect the degree of complexity of the protocadherin β-mediated neuron-neuron interaction remains to be determined. It is noteworthy that the overall gene content in the vertebrate protocadherin clusters does not seem to be correlated to their respective brain complexity. For example, while the anole, fugu and zebrafish protocadherin clusters contain 71, >77 and >107 genes, respectively [27]–[29], only 53 protocadherin genes are present in the human protocadherin cluster.

The mammalian protocadherin γ subcluster contains three divergent paralog subgroups, the γa, γb and γc, which in human, consist of 12 (*Hsγa1-12*), seven (*Hsγb1-7*) and three (*Hsγc3-5*) genes, respectively. The coelacanth protocadherin γ subcluster also contains three major paralog subgroups. However, while it is clear that the last five genes (*Lmγ20-24*) at the 3′ end of the coelacanth subcluster belong to the γc subgroup, the other two coelacanth paralog subgroups, which consist of *Lmγ1,3,4,6,7,9,11-16,19* and *Lmγ2,5,8,10,17,18*, respectively, do not seem to be directly related to any of the mammalian γa and γb subgroups [20]. The anole protocadherin γ subcluster comprises 38 genes and represents the largest γ subcluster identified to date. Our phylogenetic analysis shows that the anole γ subcluster genes also segregate into three paralog subgroups, which clearly belong to the γa (*Acγa1-10*), γb (*Acγb1-23*) and γc (*Acγc3-7*) subgroups, respectively (Fig. 3). Similar to the mammalian γa and γb subgroup genes [2], [22], the anole *Acγa1-10* and *Acγb1-23* genes are interspersed in the cluster (Fig. 1). This type of gene arrangement implies that some of the paralogs in the γa and γb subgroups might have been duplicated simultaneously as a contiguous syntenic block at some stage during evolution. Interestingly, our phylogenetic analysis shows that the coelacanth subgroup *Lmγ1,3,4,6,7,9,11-16,19* is more closely-related to mammalian and anole γa subgroups, whereas the *Lmγ2,5,8,10,17,18* subgroup is orthologous to the mammalian and anole γb subgroups [20]. Similar to their mammalian and anole counterparts, genes in these two coelacanth protocadherin subgroups also exhibit an interspersed distribution pattern, which seems to be a unique feature of the γ subcluster genes. Interestingly, no paralog subgroups analogous to γa and γb were observed in fugu and zebrafish γ subclusters [27]–[29], suggesting that γa and γb subgroups are likely to be unique to tetrapods and coelacanth.

In contrast to protocadherin genes that undergo repeated gene duplication and degeneration, the mammalian protocadherin cluster contains a subset of “ancient” genes that are less prone to gene duplication. These genes are referred to as the “c-type” protocadherin genes, which include the last two genes (*αc1-2*) at the 3′ end of the α subcluster and the last three genes (*γc3-5*) in the γ subcluster [2], [22]. Despite being located in different subclusters, these genes are phylogenetically more closely-related to each other than to other protocadherin genes in their respective subclusters [2], [22], [24]. The anole protocadherin cluster contains seven such c-type genes: two (*Acαc1* and *Acαc2*) located in the α subcluster and five (*Acγc3*-7) in the γ subcluster (Fig. 1). As shown above, the *Acαc1* and *Acαc2* genes in anole α subcluster are clearly orthologous to human *Hsαc1* and *Hsαc2*, respectively, indicating that unlike other protocadherin genes in the subcluster, the *αc1* and *αc2* seem to have never experienced gene duplication or degeneration since the divergence of reptiles and mammals. Expression studies in mammals have shown that while other protocadherin genes in the α subcluster are only expressed by selected subset of neurons, the *αc1* and *αc2* seem to be expressed by every neuron [7], [32], suggesting that they might play a key role in establishing the neural network. In the anole protocadherin γ subcluster, while *Acγc3* and *Acγc5* are clearly orthologous to human *Hsγc3* and *Hsγc5*, and coelacanth *Lmγ20* and *Lmγ23*, respectively, the *Acγc4* and *Acγc6,7* seem to have no direct orthologs in human. Instead, the anole *Acγc4* and *Acγc7* are orthologous to coelacanth *Lmγ22* and *Lmγ24*, respectively (Fig. 3, S1). No direct interspecies orthologs for anole *Acγc6*, human *Hsγc2* and *Lmγ21* were found in this analysis. Lack of direct evidence of recent gene duplication in this protocadherin subgroup suggests that the ancient protocadherin γ subcluster might have contained more c-type paralogs than any of the γ subclusters in the modern day vertebrates, and subsequent to the divergence of vertebrates, the differential gene loss, rather than gene duplication, has played a major role in the evolution of these c-type genes in the γ subcluster.

Consistent with the results of the phylogenetic analysis of constant exons of the δ subcluster (Fig. 2), phylogenetic analysis of the variable exons also showed that the single protocadherin gene in the anole δ subcluster is a direct ortholog of the coelacanth δ subcluster gene (Fig. 3). Thus, the protocadherin δ subcluster seems to be present in all non-mammalian vertebrate lineages, including reptiles, birds, amphibians, coelacanth, teleosts and cartilaginous fishes [21]. Unlike the protocadherin genes in their neighboring subclusters, none of the protocadherin δ subcluster genes seems to have undergone gene duplication. Such a stable state during evolution suggests that the protocadherin δ subcluster gene might play a critical role in establishing the neural network connections specific to non-mammalian vertebrates. The effect of the loss of this cluster in mammals is unclear.

### A model for the evolution of protocadherin cluster genes in tetrapods

**Figure 4:**
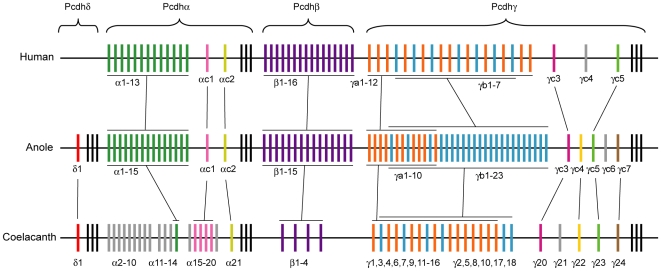
Evolutionary relationships of protocadherin clusters in coelacanth, the green anole lizard and human. Variable exons are shown as colored or grey vertical bars. The constant exons are shown as black vertical bars. Paralogs in the same subgroup or orthologs between the species are shown in the same color.

Based on the inferred phylogenetic relationships of anole, human and coelacanth protocadherin cluster genes, we propose a model for the evolution of protocadherin clusters in tetrapods (Fig. 4). In this model, we propose that repeated gene duplications and degenerations have played a predominant role in the evolution of protocadherin clusters in tetrapods. How these highly homologous and apparently redundant protocadherin paralogs affect the development and complexity of the nervous system is currently unknown. In addition, our model suggests that the paralog subgroup degeneration seem to have played an important role at the early stage of tetrapod evolution (*e.g.* during the transition from lobe-finned fishes to tetrapods), but not during the transition from reptiles to mammals. Moreover, our phylogenetic analysis supports that differential gene losses rather gene duplication play a predominant role in the evolution of protocadherin γc genes. Given the potential role of protocadherin genes in establishing the neural network, we speculate that the rapid gene turnover of protocadherin paralogs might have contributed to the adaptive evolution of the central nervous system in different tetrapod lineages. Thus, a future challenge will be to investigate how these different complements of protocadherin genes have contributed to the complexity of the nervous system in different vertebrate lineages.

## Materials and Methods

### Identification and annotation of the green anole lizard protocadherin cluster

A draft assembly of the anole genome sequences based on 6.8x coverage sequences has been generated by the Broad Institute (Broad Institute AnoCar 1.0). We identified the genomic sequence of anole protocadherin cluster by TBLASTN search of the draft assembly that is made available on the University of California, Santa Cruz (UCSC) Genome Brower (http://genome.ucsc.edu) using the amino acid sequences of mammalian protocadherin constant exons as a query. The nucleotide sequence of Scaffold_147 (2,899,420 bp), which contains the protocadherin cluster gene sequences, was retrieved from the UCSC Genome Browser. Sequencing gaps in the protocadherin cluster region were filled by PCR using anole genomic DNA as template. We could fill eighteen of the 24 gaps in the anole protocadherin cluster. The sequences corresponding to these gap regions have been submitted to GenBank under accession numbers: GQ485616-GQ485633. The remaining gaps were not amplifiable by PCR due to a high content of repetitive DNA. The annotated anole protocadherin cluster sequences have been submitted to GenBank as Third Party Annotation (accession numbers: BK006912-BK006917). Variable and constant exons of the anole protocadherin cluster and the coding exons of non-protocadherin genes flanking the anole protocadherin cluster were annotated based on GENSCAN prediction (http://genes.mit.edu/GENSCAN.html) and homology to known protein sequences in the public database (TBLASTN and BLASTX, http://blast.ncbi.nlm.nih.gov). The intron/exon splicing sites of the constant regions and the splicing sites between constant and selected variable exons in the anole protocadherin δ, α and γ subclusters were confirmed by RT-PCR using cDNA prepared from anole total brain RNA.

### Synonymous substitution analysis

Synonymous substitution rates were estimated using CODEML program in the PAML package [33]. The amino acid sequences were aligned by ClustalX and the nucleotide sequence alignments were generated based on the amino acid sequence alignment as template using RevTrans program [34]. The synonymous substitution rate was calculated as average of synonymous substitutions per codon (dS) for each branch in the gene tree of protocadherin subgroups.

### Phylogenetic analysis

The coelacanth protocadherin cluster was assembled from BAC sequences in the GenBank (accession numbers: AC150238, AC250248, and AC150308-AC150310) [20]. The human protocadherin cluster sequences were retrieved from the human genome database at the UCSC Genome Browser (http://genome.ucsc.edu). The amino acid sequences of the constant exons (see Fig. 2) or the ectodomains 1–3 (EC1-3) (see Fig. 3) of the protocadherin cluster genes from various species were aligned using ClustalW [35] as implemented in BioEdit sequence alignment editor [36] under default parameters. Only the extracellular EC1-3 sequences were used for the phylogenetic analysis because this region is less prone to gene conversion-mediated sequence homogenization, which, to some extent, would mask the phylogenetic signals [27], [29]. ModelGenerator [37] was used to deduce the best-suited amino acid substitution model for the alignments. Maximum likelihood trees were generated using PhyML [38] and displayed using NJplot (http://pbil.univ-lyon1.fr/software/njplot.html). The robustness of the tree was determined using 100 bootstrap replicates. All the trees were unrooted.

## Supporting Information

Figure S1 Phylogenetic analysis of c-type protocadherin and the protocadherin δ subcluster genes.
(0.65 MB PDF)
