# Supplementary material for: Identification and Comparative Analysis of the Protocadherin Cluster in a Reptile, the Green Anole Lizard
Source: PLoS One. 2009 Oct 29;4(10):e7614. doi: 10.1371/journal.pone.0007614 (PMC2764143; doi:10.1371/journal.pone.0007614)
Supplement: Figure S1 — Phylogenetic analysis of c-type protocadherin and the protocadherin δ subcluster genes. (0.65 MB PDF) [file pone.0007614.s001.pdf]

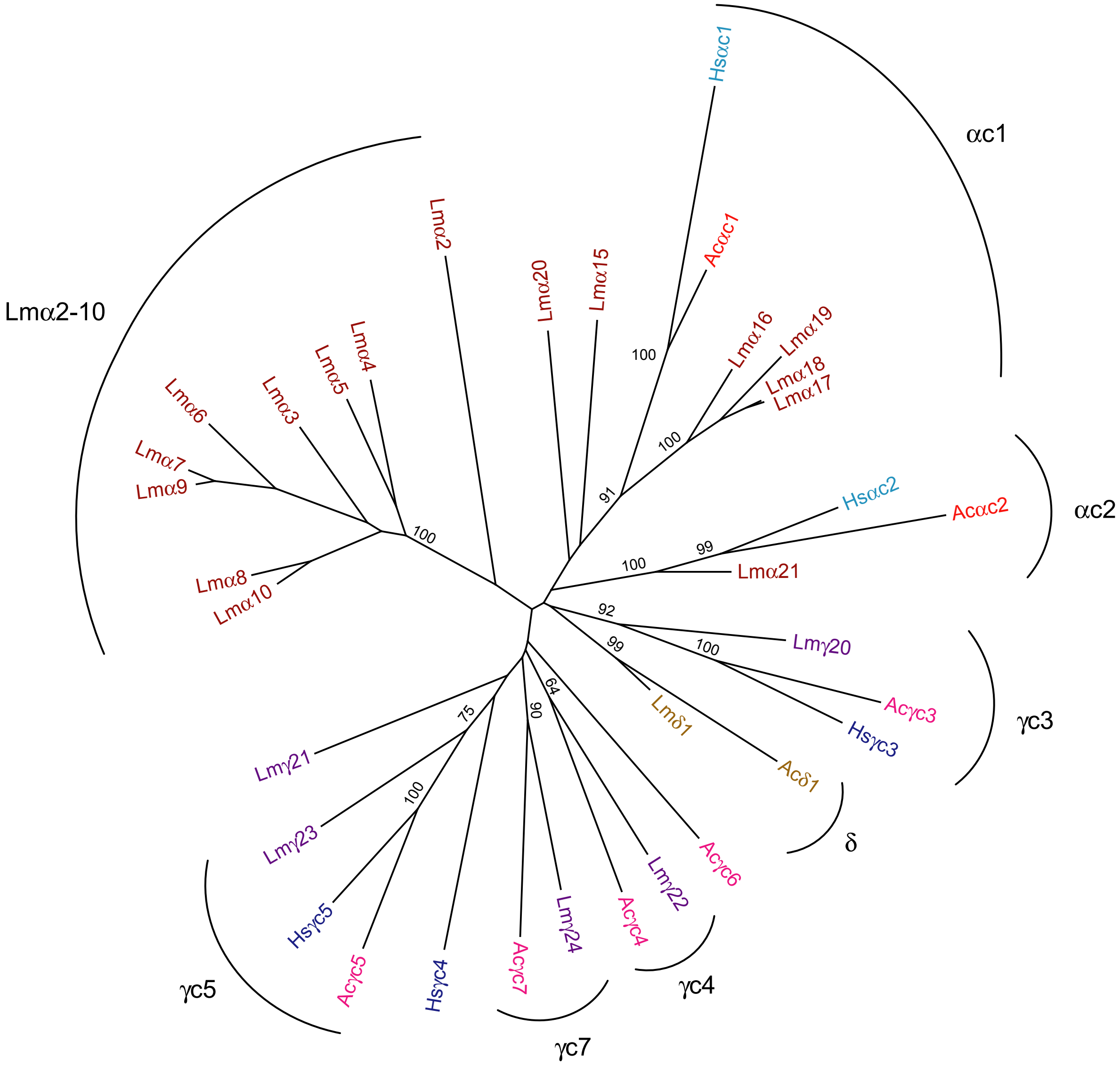

Figure S1. Phylogenetic analysis of c-type protocadherin and the protocadherin δ subcluster genes.

Amino acid sequences for the EC1-EC3 ectodomain region of anole, human and coelacanth protocadherin variable exons were aligned by ClustalW. The phylogenetic tree was constructed by the maximum-likelihood method. Genes in different protocadherin subclusters are indicated by color. Numbers at the nodes are the percentage of bootstrap values of 100 replicates. The tree is unrooted.
